# Supplementary material for: Knockout of circRNAs by base editing back-splice sites of circularized exons
Source: Genome Biol. 2022 Jan 10;23:16. doi: 10.1186/s13059-021-02563-0 (PMC8744252; doi:10.1186/s13059-021-02563-0)
Supplement: Supplementary file 1 — Additional file 1: Supplementary figures. Fig. S1 Base changes at back-splice sites by BE4max or eBE-S3 (Related to Fig. 2). Fig. S2 Base changes at back-splice sites by ABEmax (Related to Fig. 2). Fig. S3 Validation of circRALY-nov and circCAMK1D-nov (Related to Fig. 4). Fig. S4 Thirteen circRNA KO by base changes (Related to Fig. 5). Fig. S5 Functional analysis of circZNF292-nov and circRALY-nov (Related to Fig. 5). Fig. S6 Overview of current strategies for circRNA knockout (Related to Fig. 6). Fig. S7 Examination of editing ratios of on-target sites and mutation ratios at selected gRNA-dependent off-target sites in CDR1as/ciRS-7 KO and negative control monoclones [file 13059_2021_2563_MOESM1_ESM.docx]

**Supplementary Information**

Supplemental figures and figure legends

Additional file 2: Table S1

List of high-confidence circRNAs in 293FT Cells. High-confidence circRNAs was determined from ribo–, poly(A)– and RNaseR-treated RNA-seq in 293FT cells, shown by circRNA location, strand, gene symbol, transcript ID, included exons, FPB in ribo–, p(A)– and RNaseR-treated samples, whether can be targeted by hA3A-eBE-Y130F or ABEmax, whether have predominantly bs-exons, and whether have novel bs-exons.

Additional file 3: Table S2

List of oligonucleotides and primer sequences used in this study. (A) Sequences of oligonucleotides used in sgRNA constructs. (B) On-target primer sequences used in genomic DNA amplification. (C) The gRNA-dependent off-target primer sequences used in genomic DNA amplification. (D) Primer sequences used in RT-qPCR and RT-PCR analysis. (E) Primer sequences used in NB. (F) Sequences of oligonucleotides used in shRNA constructs.

**
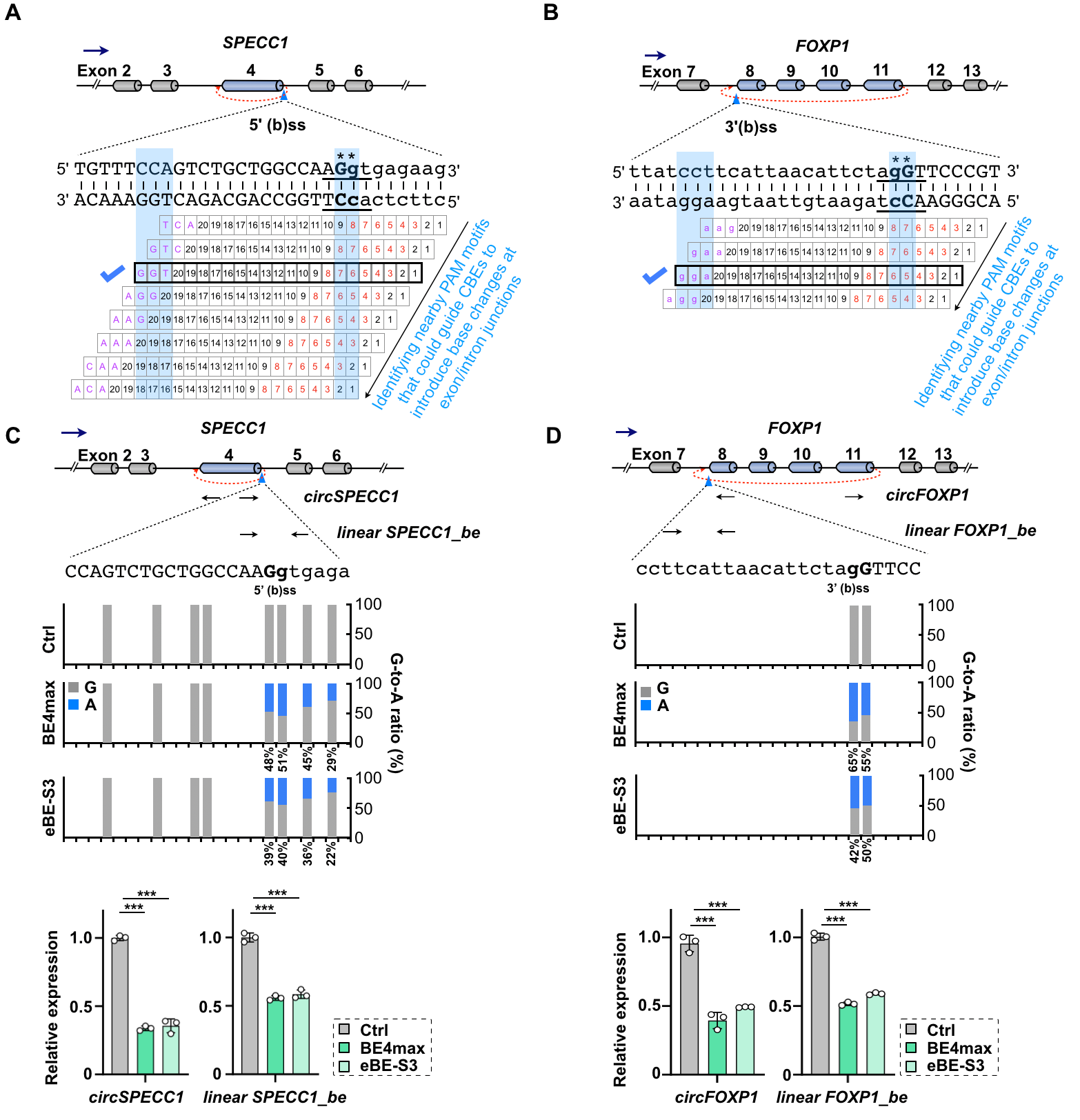
**

**Fig. S1 Base changes at back-splice sites by BE4max or eBE-S3 (Related to Fig. 2)**

(A and B) The diagram for judging whether 5'/3' (b)ss site is potentially targetable by CBEs. If there are PAM sequences that fit cytosine(s) that are base-paired at the complementary strand to guanine(s) at exon/intron junctions of back-splice sites into the editing window of hA3A-eBE-Y130F. * indicates targeted bases of 5'/3' (back-) splice signal. For sgRNA, PAM sequences were labeled in purple, and bases in the editing window were labeled in red.

(C) Base changes of 5' (b)ss of *circSPECC1* by both BE4max and eBE-S3 repressed both back-splice and canonical splice. Top, schematic of targeted gene organization. Back-spliced exon was highlighted by blue bar. Context sequences of targeted 5'/3' (b)ss were shown by a, t, c and g for intron or by A, T, C and G for exon; Middle, G-to-A base change ratio at targeted 5'/3' (b)ss of back-spliced exons; Bottom, evaluation of back-splice and splice changes by RT-qPCR using primers labeled on the top.

(D) Base changes of 3' (b)ss of *circFOXP1* by both BE4max and eBE-S3 repressed both back-splice and canonical splice. Refer to (A) for details.

(C-D) Error bar represents SD from three independent replicates. ∗∗∗, *P* < 0.001; Student’s t test.


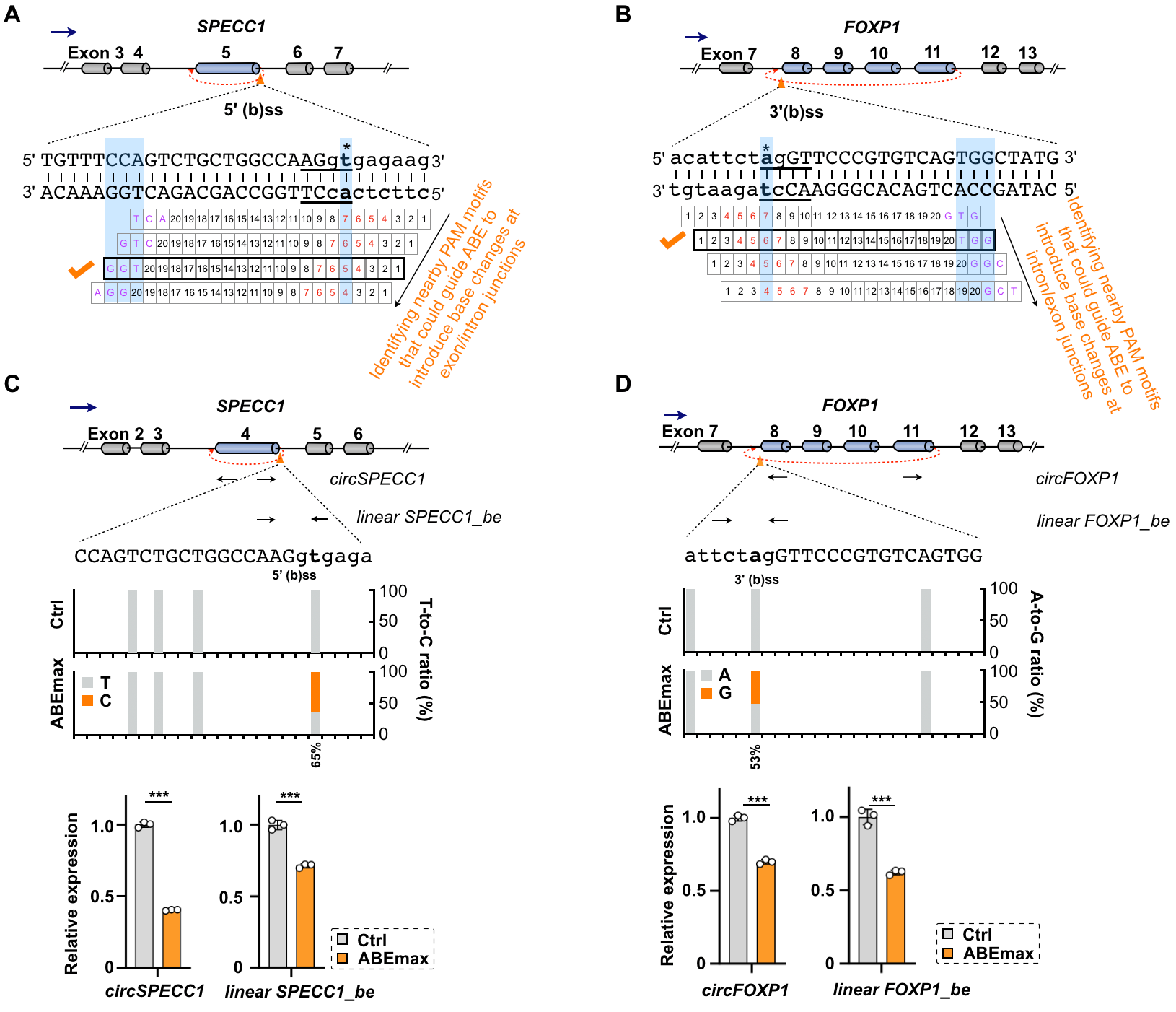


**Fig. S2 Base changes at back-splice sites by ABEmax (Related to Fig. 2)**

(A and B) The diagram for judging whether 5'/3' (b)ss site is potentially targetable by ABEs. If there are PAM sequences that fit adenosine(s) at 5' (b)ss or at the complementary strand at 3' (b)ss, into the editing window of ABEmax. * indicates targeted bases of 5'/3' (back-) splice signal. For sgRNA, PAM sequences were labeled in purple, and bases in the editing window were labeled in red.

(C and D) Mutation of 5' (b)ss of *circSPECC1*or *circFNTA* by a highly efficient adenine base editor, ABEmax, repressed both back-splice and canonical splice. Top, schematic of targeted gene organization. Back-spliced exon was highlighted by blue bar. Context sequences of targeted 5' (b)ss were shown by a, t, c and g for intron or by A, T, C and G for exon; Middle, base change ratio at targeted 5' (b)ss of back-spliced exons; Bottom, evaluation of back-splice and splice changes by RT-qPCR using primers labeled on the top. for details. Error bar represents SD from three independent replicates. ∗∗, *P* < 0.01; ∗∗∗, *P* < 0.001; Student’s t test.


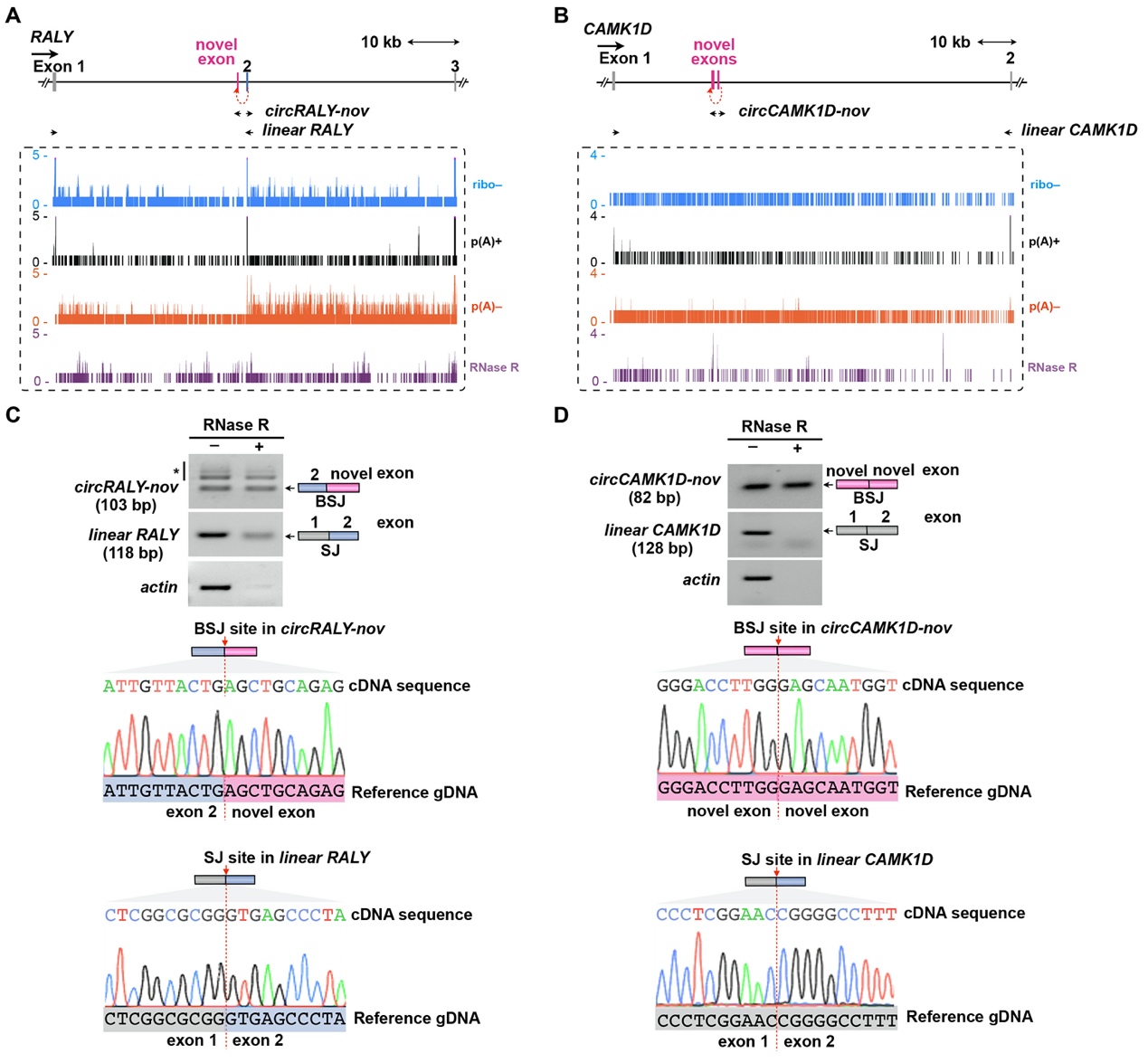


**Fig. S3 Validation of *circRALY-nov* and *circCAMK1D-nov* (Related to Fig. 4)**

(A) Schematic of the partial *RALY* gene organization and mapped RNA-seq signals in 293FT cells. A previously-unannotated exon (pink bar) between annotated exons 1 and 2 in the RALY gene locus was suggested to be back-spliced with exon 2 to form circRALY-nov in 293FT cells.

(B) Schematic of the partial *CAMK1D* gene organization and mapped RNA-seq signals in 293FT cells. Two previously-unannotated exons (pink bar) between annotated exons 1 and 2 in the *CAMK1D* gene locus were suggested to be back-spliced to form *circCAMK1D-nov* in 293FT cells.

(C) A previously-unannotated exon in the *RALY* gene locus was predominantly back-spliced for circRNA biogenesis, but barely spliced in cognate linear RNAs. Top, amplification of the back-splice event between exon 2 and the previously-unannotated exon in the *RALY* gene locus and the canonical splice event between exons 1 and 2. Of note, the back-splice event that suggests for the expression of *circRALY-nov* was retained with RNase R treatment. Differently, the canonical splice (between exons 1 and 2) events that suggest for the expression of linear *RALY* RNA(s) or *actin* mRNA were largely degraded with RNase R treatment. Validation of the back-splice (middle) and the canonical splice (bottom) events in the *RALY* gene locus was performed by Sanger sequencing. Corresponding BSJ and SJ sites were confirmed from amplified back-spliced or canonical spliced cDNAs, shown in middle. * indicates amplified product from *circRALY-nov*, possibly due to rolling PCR of examined *circRALY-nov*.

(D) Two previously-unannotated exons in the *CAMK1D* gene locus were predominantly back-spliced for circRNA biogenesis, but barely spliced in cognate linear RNAs. Refer to (C) for details.


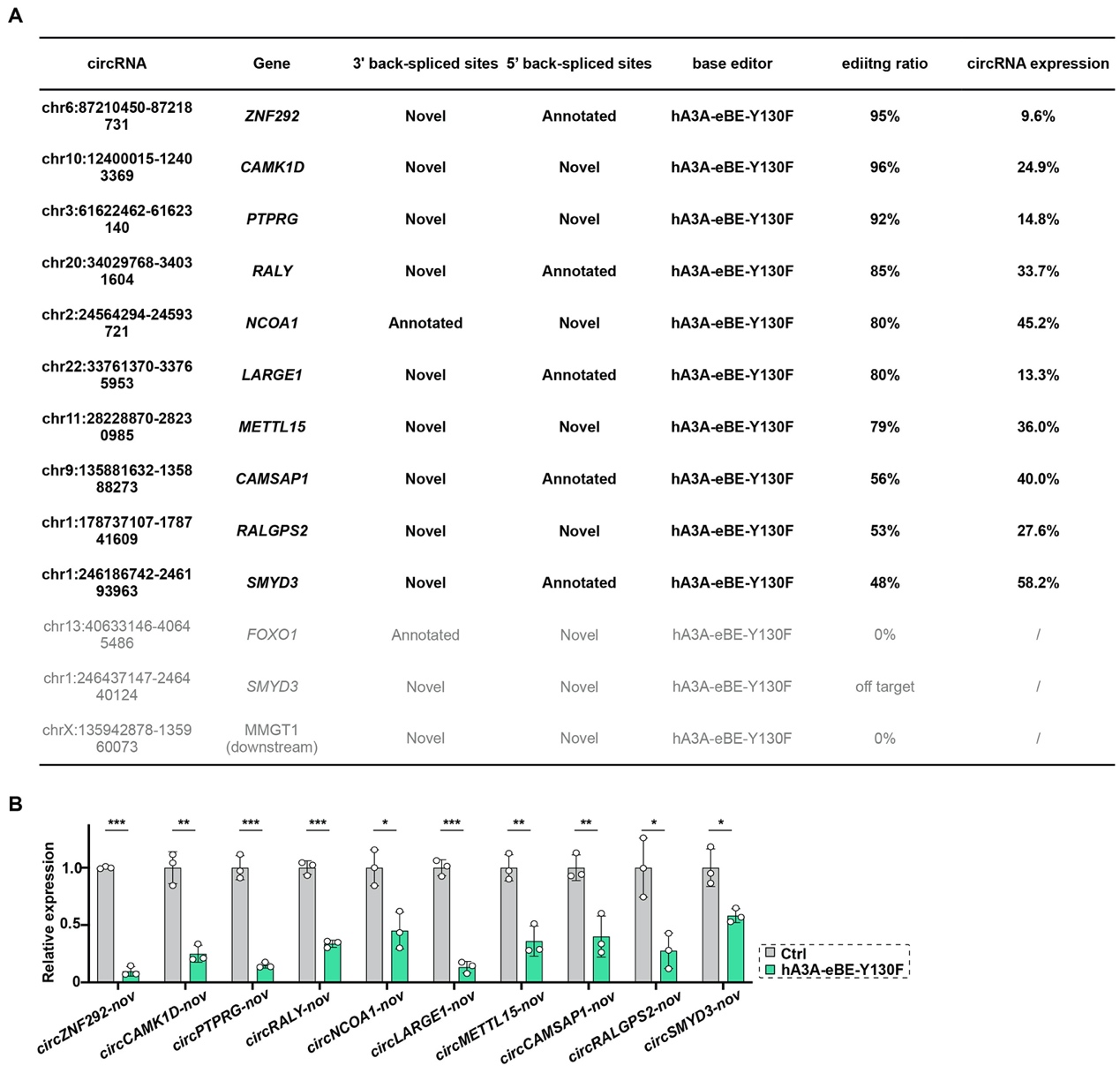


**Fig. S4 Thirteen circRNA KO by base changes (Related to Fig. 5)**

(A) The table lists of circRNA information and base editing results, including localization, gene name, annotation information of back-splice sites, base editor, editing ratio and circRNA expression level.

(B) Ten circRNAs are knocked down by BE, as revealed by RT-qPCR.


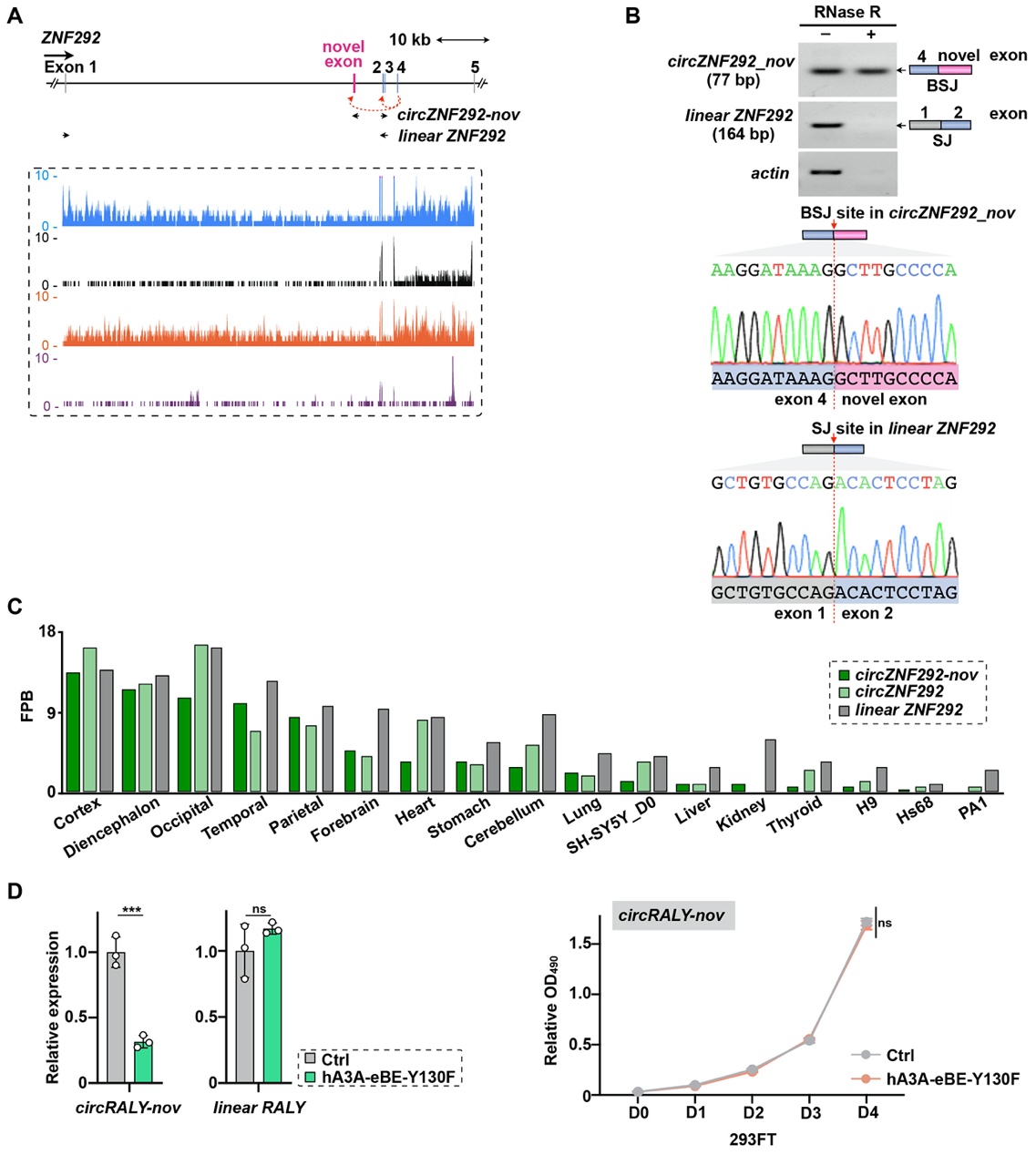


**Fig. S5 Functional analysis of *circZNF292-nov* and *circRALY-nov* (Related to Fig. 5)**

(A) Schematic of the partial *ZNF292* gene organization and mapped RNA-seq signals in 293FT cells. In addition to a back-splice between exon 4 and 2, a previously-unannotated exon (pink bar) between annotated exons 1 and 2 in the *ZNF292* gene locus was suggested to be back-spliced with exon 4 to form *circZNF292-nov* in 293FT cells.

(B) A previously-unannotated exon in the *ZNF292* gene locus was predominantly back-spliced for circRNA biogenesis, but barely spliced in cognate linear RNAs. Top, amplification of the back-splice event between exon 4 and the previously-unannotated exon in the *ZNF292* gene locus and the canonical splice event between exons 1 and 2. Of note, the back-splice event that suggests for the expression of *circRALY-nov* was retained with RNase R treatment. Differently, the canonical splice (between exons 1 and 2) events that suggest for the expression of linear *ZNF292* RNA(s) or *actin* mRNA were largely degraded with RNase R treatment. Validation of the back-splice (middle) and the canonical splice (bottom) events in the *ZNF292* gene locus was performed by Sanger sequencing. Corresponding BSJ and SJ sites were confirmed from amplified back-spliced or canonical spliced cDNAs, shown in middle.

(C) The expression levels of *circZNF292-nov*, *circZNF292* and linear *ZNF292* across different tissues/cell lines.

(D) Left, evaluation of back-splice and splice changes of *RALY* by RT-qPCR using primers labeled at (S3). Since the identified novel exon was only back-spliced in *circRALY-nov*, base changes by hA3A-eBE-Y130F at its 3' bss back-splice site only affect back-splice of *circRALY-nov*, but not canonical splice for linear *RALY* RNA(s) with annotated exons; Right, repression of *circRALY-nov* had no effect on 293FT cell proliferation, as revealed by MTT assays. Error bar represents SD from at least three independent replicates. ns, not significant; ∗∗∗, *P* < 0.001; Student’s t test.


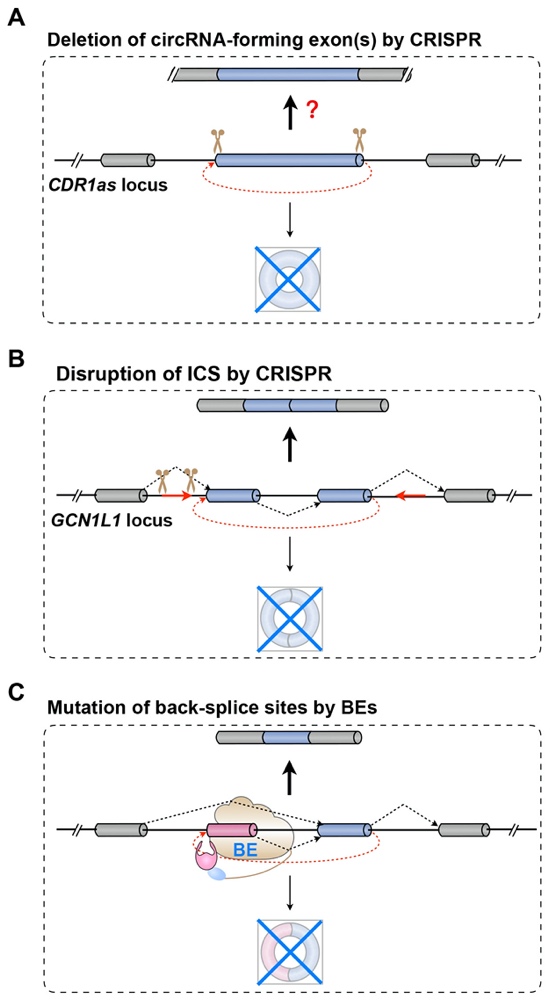


**Fig. S6 Overview of current strategies for circRNA knockout (Related to Fig. 6)**

(A) CircRNA knockout is achieved by directly removing the circRNA-formed exon(s) by CRISPR/Cas9 system, such as *CDR1as*/*ciRS-7* KO in mouse.

(B) Disruption of the pairing between intronic ICS flanking circRNA-forming exons by CRISPR/Cas9 system to minimize circRNA generation with little effect on the expression of residing protein-coding gene, such as *circGCN1L1* KO in human PA1 cells.

(C) Mutation of back-splice sites by BEs to disrupt back-splice signals, especially at back-splice sites of predominantly back-spliced novel exons to repress circRNA expression specifically.


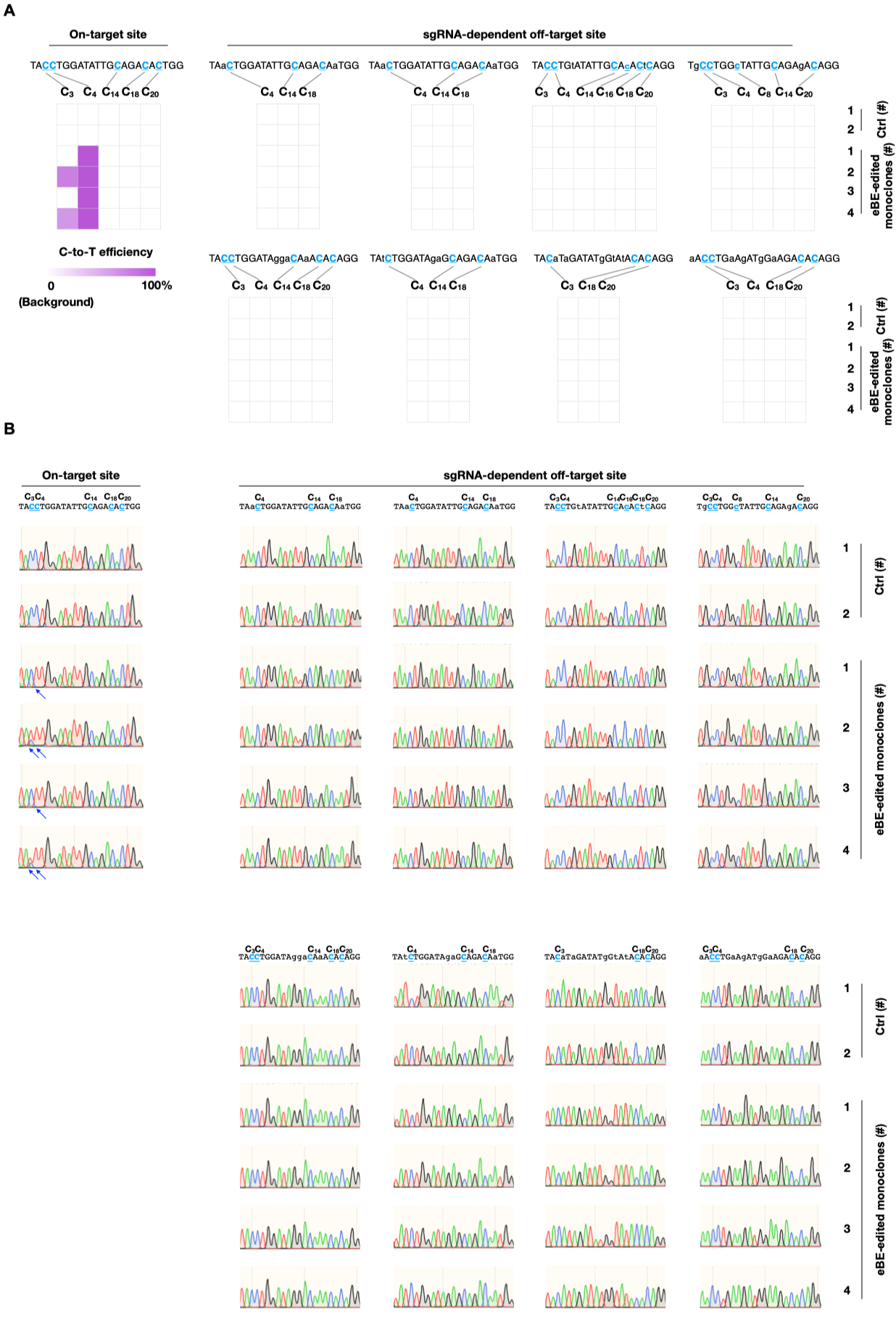


**Fig. S7 Examination of editing ratios of on-target sites and mutation ratios at selected gRNA-dependent off-target sites in *CDR1as*/*ciRS-7* KO and negative control monoclones.**

(A) Evaluation of editing or mutation frequencies induced by hA3A-eBE-Y130F at on-target sites or sgRNA-dependent off-target sites with 2, 3, 4 or 5 mismatches in four *CDR1as*/*ciRS-7* KO monoclones. Two negative control monoclones were used for comparison. Cytosine bases within on-target or off-target site sequences are shown in blue with underline (setting the base distal to the PAM as position 1). C-to-T editing/mutation ratios were calculated from Sanger sequencing shown in (B). Lowercase letters indicate the mismatch base positions at predicted gRNA-dependent off-target sites by the previously-published Cas-OFFinder method [60]. Of note, efficient on-target editing was examined at on-target sites in all four monoclones, but not the negative control monoclones. Meanwhile, no extra mutation could be found in all examined sgRNA-dependent off-target sites with 2, 3, 4 or 5 mismatches.

(B) Original Sanger sequencing results to show C-to-T editings at on-target (left) or mutations at sgRNA-dependent off-target (right) sites.
